# Public Health Education

**Published:** 1921-10

**Authors:** Arthur Newsholme


### PUBLIC HEALTH EDUCATION.

By Sir Arthur Newsholme, K.C.B., M.D., F.R.C.P.*

Although nearly three years have passed since there was formal cessation of war, we are all of us still wrestling with post-bellum problems of adjustment to vital changes in social outlook. It is not surprising that there should be unrest and unsettlement after such titanic events. A large part of the manhood of the United States was under arms in the later stages of the war, and for the various parts of the British Empire I may recall figures which help to explain present British conditions, while giving reasonable ground for national pride. In Great Britain alone five and two-third million men volunteered for military or naval service before such service became compulsory, and three millions more tried to enlist, but were too old, or physically unfit, or were needed for other work. The following figures may be quoted, showing, for different parts of the Empire, the proportion of the total white population of each country who shared in the fighting: England, 24.02 per cent.; Wales, 21.52 per cent.; Scotland, 23.71 per cent.; Ireland, 6.14 per cent.; Canada, 13.48 per cent.; Australia, 13.41 per cent.; New Zealand, 19.35 per cent.; South Africa, 11.12 per cent.

The severity of the struggle is demonstrated by the fact that the British casualties numbered over three millions, of whom nearly two and a-half millions were from the United Kingdom. In the British Empire the killed and wounded numbered 4.51 per cent. of its total white population; in France, 4.31 per cent.; in Serbia, 3.56 per cent.; in Belgium, 0.78 per cent.; in the United States, 0.22 per cent.

There is satisfaction as well as sadness in these figures. They give the lie to the assumption that our manhood was decadent, either physically or in spirit. They prove that under the overpowering impulse of patriotism men are prepared to sacrifice life or limb or reason for their country. They have shown that the two great branches of the Anglo-Saxon world were ready to fight side by side against militarism and on behalf of freedom and idealism. Incidentally, they revealed the practicability in emergency of women undertaking a large share of men's work, and their willingness to make sacrifices of health only less than those of the men.

From a social point of view a great incidental gain of war has been in the improved physique and health of most of the returned soldiers. It would be a magnificent thing for our future well-being if every adolescent could secure between the ages of sixteen and twenty a month's annual physical training and discipline, during which period he would enjoy an admirable holiday, receive intellectual refreshment and guidance, and gain a satisfactory realisation of his physical possibilities.

### Prevalence of Physical Defects.

The physical examination of recruits, however, showed that approximately one-third of their number had physical disabilities of greater or less severity. Many of these defects were of a minor character, such as flat feet or varicose veins, but a large proportion were more serious; and an inadequate realisation of the importance of health, and of the extent to which disease may be prevented or cured, must be inferred in view of the enormous proportion of our total adult population which, when sampled, was shown to be thus handicapped for civilian, as well as for military, efficiency. The evidence thus afforded merely supplements that known to our physicians and health-officers of the prevalence of preventable disease and of an unnecessarily low standard of health, which, although avoidable, is not avoided.

We may not unprofitably be asked, especially those of us who have not participated actively in the world war, what should be our share in the world's work of rehabilitation? Are we taking our part not only in securing international peace for the future, but also in combating the horrors of peace, which in the aggregate are more serious than those of war? The ideal means for the prevention of war is a United States of the world, with federal control of the world's constabulary, and then we might realise the millennium pictured by William Watson of the time when will come that

> morn divine
> When nations shall as forests grow,
> Wherein the oak hates not the pine,
> Nor beeches wish the cedars woe,
> But all in their unlikeness blend
> Confederate to one glorious end.

### The Amount of Preventable Disease.

A healthy population is needed for national efficiency and economy, and to enable each person to realise his or her possibilities of success and happiness. It is not sufficiently realised that at least one-half of our present mortality is avoidable—*i.e.*, could be postponed to old age when death becomes a normal event of life. With this unnecessary mortality goes an incredibly large amount of avoidable sickness, and the object of public health is to prevent this sickness and to enhance the average standard of health.

I need not illustrate this point in detail. Tuberculosis causes one-tenth of the deaths from all causes, and in the best working years of life one out of every three deaths from all causes is due to this disease. It is the largest producer of dependent widows and orphans. Syphilis is probably as great a cause of mortality and disease as tuberculosis. One-third of the blindness of young children is caused by it. Were it to disappear our lunatic asylums would be robbed of one of every ten of their inmates. Syphilis is the most common cause of premature arterial degeneration and apoplexy. The disabilities caused in the State of Virginia by malaria, by hookworm, and by trachoma are well known.

* Part of an Address prepared for delivery to the University of Virginia, April, 1921.

**Public Health Education—(*cont.*).**

In the application of remedial measures, what are our essential needs? They may be summed up under two heads: (1) The teaching and application of knowledge already available; and (2) extensions of our present knowledge, not only by means of laboratory investigations, but also by "field" investigations, by social and sanitary surveys.

In both of these respects the university, and not only the medical section of it, forms a most appropriate rallying ground for further effort.

THE STUDY OF PREVENTION.

That the university is the right sphere for research will be at once admitted. The need for the association of advanced teaching with research in any subject is becoming more widely realised. The professor whose whole time is occupied in teaching is likely to lose freshness and to become a non-inspiring guide for his students. Furthermore, the university is the seat of many allied branches of learning, and the research worker in one subject can more easily than elsewhere obtain the valuable assistance of other workers. This is especially true for hygiene and medicine, and at this and other universities we may confidently expect to receive new light on the dark corners of preventive medicine which remain. The problems of cancer, of the prevention of catarrhal infections, of means for reducing the fatality of pneumonia, at once occur to the mind as among the regions in which we still grope in almost mediæval darkness.

Of surveys we have already much experience. They do admirable service in drawing attention to serious evils and in indicating the lines along which these evils can be successfully combated. Many such surveys have been made on both sides of the Atlantic. It was the surveys made by the medical inspectors appointed by Edwin Chadwick in the early 'forties of the last century which gave the impetus to early English sanitary legislation and local reform. The surveys would probably have had as little effect in securing prompt reform as many subsequent surveys have had, but for great epidemics of Asiatic cholera and fever and the collection of the death returns showing the enormous losses of life, which roused Parliament to the need for action. Panic in the past has been a great friend of public health reform; and it is one of the many signs of progress that, in the absence of epidemics of preventable diseases on the scale of former decades, progress is now being made in their prevention.

Probably even more valuable than surveys in securing further knowledge of preventive measures and the application of existing knowledge has been experimental work on a small scale, often started by voluntary agencies before being taken up by public health authorities. This is well illustrated in child-welfare work and in tuberculosis work. These activities have most often begun under voluntary auspices. Voluntary societies, with their greater freedom, can make experiments and demonstrate the feasibility of new lines of work more easily than official agencies, and the value of not a little of our present official public health activities has been thus demonstrated.

It is not likely that in the future voluntary agencies will greatly diminish their activities, and there is no reason why they should not continue as feeders and stimuli of official public health work. In the two instances named there remain, in most communities, both voluntary and official agencies side by side, and, although sometimes this has meant some measure of inco-ordination and considerable waste of energy, it cannot be said that in any large community the total efforts made by both agencies are commensurate with the magnitude of the problem. Volunteer work has usually been the pioneer in extension of public health work on its personal side, and it may be stated generally that demonstration of such work on a small scale, thus showing the need for it and the practicability of meeting this need, is a more efficient method of progress than an elaborate and complete paper scheme which fails to materialise or than an annual "drive" for special work, the impetus of which is almost exhausted in the drive itself and in paying secretarial salaries, leaving little money or energy for the work itself.

In regard to education bearing on health, the university and the detached medical school can be of inestimable service; and this ideal is to a large extent realised in the University of Virginia. First there is the great influence of its medical school. Not only is this a great centre for the scientific training of the medical practitioners of the future, but in the fulfilment of this aim centres have been established here for the treatment of venereal diseases and tuberculosis, and you have a general hospital supplying a large part of West Virginia.

This is a practical illustration of prevention by means of treatment, one of the most important branches of preventive medicine. Illustrations of this means of prevention are familiar to us all. In syphilis, in tuberculosis, in malaria, in hookworm disease, in rickets, treatment forms an important means of preventing disease in others and of curtailing the duration of disease in the patient under treatment. A remarkable example of prevention of goitre has recently been demonstrated in States bordering on the Great Lakes, where it has been found that the administration of sodium iodide removes goitrous swellings in school-children, that it has the same effect experimentally in dogs in the same area, and that the same means can be employed for preventing thyroid enlargement. The value of cod-liver oil in the prevention, as well as in the treatment, of rickets has been too recently demonstrated to need more than passing reference.

The primary object of the clinics at universities and medical schools is the practical teaching of medical students. They may be, and probably already are, in part made a means of initiating and daily deepening the conviction in the minds of these students of the need for considering prevention in treatment. Satisfactory treatment can only follow on accurate diagnosis of each patient's case, social as well as medical; and diagnosis implies an investi-

**Public Health Education—(*cont.*).**

gation into causation, again social as well as medical; there is thus opened up for each physician the possibility, nay, the duty, of becoming a health officer to the full extent of the range of his medical practice.

FAMILY DIAGNOSES.

Once this idea penetrates thoroughly into the conscience of every physician there will be much closer and more constant co-operation between private physicians and medical officials than is now secured. On the one hand, this will mean more prompt certification of infectious diseases, more complete and accurate certification of deaths; and, on the other hand, it will lead inevitably also to family as well as personal diagnosis. A patient consults a physician for illness which is diagnosed as tuberculosis, or syphilis, or hookworm disease, or malaria, or trachoma. In all these, and in many other, instances the possibility, indeed, the likelihood, of similar infection of other members of the same household is involved. Is the physician's responsibility fulfilled when he has prescribed for his patient? Surely not. He may claim that in going further he lays himself open to the accusation of "touting for business," and it must be confessed that our present system of payment by fee per consultation or visit makes it difficult for the physician to offer extended activity. This is one of the strongest arguments for reform in our system of medical attendance and for the substitution for fees for individual visits of annual payment for medical service for an entire family. Such a substituted plan would fail to serve the interests of preventive medicine unless it included a periodical—say, three-monthly—interview with each member of the family, whether ill or well. Failing such reconstructive measures, the tactful and conscientious physician can, even under present circumstances, become a most important public health missionary. Often he is already so, but for the masses of the population it must be confessed that this ideal is remote.

TEACHING OF PREVENTIVE MEDICINE.

The family physician's potentialities for communal service are almost unlimited. It is the function of the Medical School to ensure that this power for good is appreciated by every student, and that the teaching of every medical subject is organised with a view to prevention, as well as treatment. In Great Britain special training for those wishing to obtain public-health posts has been organised; and medical officers of health for all considerable communities are now required to have had a nine months' special training in public health, including the daily work of a medical officer of health, and to have obtained a diploma in public health from a university or other authorised examining body at the end of this training. There are beginnings of similar special training in the United States, and I have little doubt that ere many years have passed progressive States will have made it a condition of holding a public-health appointment that the recipient is possessed of special evidence of training and experience in his duties.

I have referred to the education of medical students and physicians in preventive medicine, and to the special training needed for health officers. I have also briefly indicated how small centres for consultation and treatment may be made valuable means of inculcating the principles and practice of health. There is needed, quite as much as training of the physicians and health officers of the future, the education in health matters of the general population, and especially of the representatives elected by the public to control local and State administration. How is this to be secured? It is already being provided spasmodically and sporadically, through voluntary agencies and to some extent by health education campaigns organised by health officers; and I do not desire to minimise the value of this work. There is little doubt, however, that its effect is absurdly small compared with the effort and money expended on it.

BAD TYPES OF PUBLICITY.

Before considering what better lines of education are practicable, it is only fair to state that much of the "publicity" propagandism has failed because of its unscientific extravagance of statement. On short view it may be possible to secure a modicum of action by highly coloured descriptions of an evil, by absurdly exaggerated promises of results of proposed action against this evil, or by foolish claims in respect of some very partial activity; but in the long run exaggeration is an impediment to real and especially to steady progress. As Dr. C. V. Chapin has put it, referring to the publicity man: "For the sake of those who come after, stop filling your columns with tommy-rot, hot air and dope. . . . Better pay your publicity man for doing nothing than for writing something which is not so. Careless writing betokens the lazy writer. Seek diligently the truth and faithfully publish it."

Our knowledge of preventive medicine, in relation to many diseases, has become so exact that there is no excuse for teaching—as has been done largely in the past—what may need subsequently to be unlearned. Our knowledge of the different methods of interrupting the life history of the malaria plasmodium is ample to enable exact preventive measures to be taken; the risks of plague can be obviated through our knowledge of the part borne by the rat in its spread; yellow fever similarly need no longer be a source of alarm; typhoid fever and dysentery can be prevented to the exact extent to which we are prepared to undertake the well-known measures of sanitation and of control of carriers now available; tuberculosis can be reduced to a fraction of its present amount by undertaking well-known measures for minimising infection and for removing conditions which favour infection; and venereal diseases similarly can be brought under control by the simultaneous universal application of the social and medical measures which will ensure this end. The above does not exhaust the

**Public Health Education—(*cont.*).**

list of preventable diseases; but it is safe to state that the diseases just enumerated cause one out of five total deaths in the community, showing what a firm and extensive basis we possess for immediate work.

The Reason of Inaction.

As we possess this knowledge, why is it not applied? Disease in any community is always more costly than the measures required for its control. This statement is axiomatic. No person knowing the facts can doubt its accuracy. The reason is that the knowledge is not really possessed by the majority of the community, or, where the facts are known, they have never become part and parcel of the deepest convictions of mankind. Theologians have distinguished between intellectual acquiescence in the tenets of religion, and the living faith which leads to a saintly life. The position as regards sanitary knowledge is analogous to this. Were it otherwise one would expect, and even hope, that not many members of our governing bodies, central or local, could enjoy comfortable sleep at night, in view of their terrible responsibility for the continuance of sanitary evils and for the lack of sanitary provisions, which result in an enormous mass of avoidable sickness and mortality.

This view appears to me to have fundamental importance. We are constantly being urged to educate, educate! But before education can be availed of more disinterested as well as more intelligent citizens are required. Education means something more and something more important than interpreting past experiences as a guide to future conduct. The educator, as it has been well said, is an artist creating something which hitherto was without form. He is, it must be remembered, a teacher, not a miracle worker, and he must wait on and aid the development of the human mind.

The basic requirement is to train the behaviour of the individual under the varying circumstances of life. Such training must be begun early if it is to have its full effect; and an essential part of such training, as educators well know, consists in cultivating the capacity to abstain from present pleasure for the sake of future benefit. "Drives" to influence the public opinion of the adult population calculated to act as does a persistent and striking advertisement in securing larger sales of a patent pill or soap, and to secure an increased allotment of funds from public sources for sanitary work, are necessary evils; but to secure permanent and steady improvement the saving grace of an altered outlook on life on the part of the majority of the community is needed, and our chief hope for this lies with the young.

It is through our university and scholastic training, and through the higher outlook of the parents of the next generation to be thus secured, that success will be achieved; and it is lamentable in this connection that the emoluments of so many university teachers are discreditably inadequate. We are still, to a large extent, asking our educators to "make bricks without straw."

This may appear to be wandering far from the subject of public health, but the formation of character, more than the cultivation of intellectual powers, or the acquisition of knowledge, is the main desideratum in education, and especially in sanitary education.

Let me give two illustrations which will make my point clearer. Even before the Great War, and still more during it and since its cessation, we have been brought to a fuller appreciation of the importance of venereal diseases and of tuberculosis as the two greatest enemies to the health efficiency and happiness of mankind. How are these to be controlled? For the first of these, the measures which are being adopted on an inadequate scale are well known. Free laboratory aids to diagnosis are provided; the notification of cases to an uncertain extent is secured; clinics for the treatment of patients are provided, thus aiding the curtailment of infectivity; a limited amount of "follow-up" work, with discovery of sources of infection is practicable; disorderly houses are being partially suppressed and street solicitation diminished; infectious women and men in prison, in some States, are detained until free from infection; and educational work of various kinds is being undertaken. This is most of it effective work, though hitherto in no community known to me has it been carried out to the full extent of its potentiality for good.

It is with anti-venereal educational work that we are particularly concerned at the moment. It commonly takes the form of free distribution of literature and of addresses to special groups on sex hygiene and on the dangers of venereal disease. The showing of lurid pictures of such diseases will, I think, sometimes turn the balance in a young man against exposing himself to infection, though there is no evidence of a smaller amount of these diseases in medical than in other students. Young men are apt to despise risks; and a much more successful plea with them is based on the lack of chivalry involved towards the woman, and on the terrible possible consequences for wife and children in the future. To lay chief stress, as is done in some programmes, on physical prophylactic measures at the time of exposure is to lean on a bruised reed, and to belittle the moral force of other arguments.

But behind the promiscuity, which is the chief source of venereal disease, what are the factors leading to exposure to risk? We have to reckon with a chief passion of humanity, and are furthermore concerned with the whole problem of self-control. This cannot always be successfully taught in the presence of an urgent temptation. It should have been built up from earliest infancy. The mere radiation of affection on a child does not train it to unselfishness and moral restraint. For these, steady and persistent discipline, first and most important, parental from earliest infancy, and next scholastic, are necessary. Play itself is one of the important agencies in assisting the development of self-control. It is an essential stage in the growth of character. In such games as football, and in most combined games, self-control and self-subordi-

**Public Health Education—(*cont.*).**

nation to the group-welfare are an essential element; and whether in play or apart from play, the future character of the child is the resultant of the systematised voluntary actions of life day by day. Were this law intelligently realised by parents and teachers and training directed to this end, how different might be the outlook for the world! Disease, which depends more on selfishness and carelessness than on actual elementary ignorance, would shrink in dimensions, and the general sum of human happiness would be vastly increased. The child is the future of the race. The early life of childhood, even before school age is reached, is the growing point of society; and it is on the safeguarding and guidance of this point that our main hope of success for the next generation, physically and morally, lies.

This is especially so in regard to venereal diseases, the circumstances producing which strike at the root of family life. It is so also to a less obvious extent in the control of tuberculosis. Although with gradually improving habits and more satisfactory treatment of cases this disease is declining, it is still, as we have seen, one of the two or three chief causes of disablement and mortality in our midst. Why is it not being brought more rapidly under control? A full statement on this point would occupy more space than can be given in this paper. The slow progress is not due to our inadequate knowledge of the causation of this disease. It is due to our not having attempted to carry out all the known measures against this disease on a scale which is commensurate with the evil. Some of these measures involve a carefulness in the personal habits of patients, a conscientious regard to elementary precautions, which patients are seldom prepared to carry out. In particular they involve abstinence from caressing of young children and other means for preventing in early life large-dosed infection. Here again we appear to be "up against" the need for a higher standard of conduct as well as a mere intellectual knowledge of the safeguards which are required. That improvement in these respects will occur, and that the rate of decline of tuberculosis will become accelerated I have no doubt; but meanwhile, while working to this end, let us note that what is needed is not only additional knowledge of this disease, but especially extended co-operative effort on the part of every member of the community to utilise our present knowledge.

### The School as a Centre of Public Health Education.

The medical inspection of school-children, the discovery of defects and disease, and the efforts to secure remedial measures form a most valuable means of hygienic education of parents. But I am more concerned for the moment in the school and the teacher as agents for training the children themselves in self-discipline and in hygienic habits which will continue to operate through life. The teacher himself should be thoroughly grounded in elementary hygiene, and the psychology of the young mind; and he should appreciate the importance of making the drinking-water arrangements, school meals, washing appliances, and especially toilet provisions the means of inculcating healthy habits. In many country districts in the United States there are no toilet arrangements in scattered homes, sometimes not even in schools. Apart from risks of hookworm disease and typhoid fever thus incurred, it is essential that the school in these matters should always be the model of what the home should be. Here is an ample field for practical example leading to the prevention of disease.

### The Public Health Nurse.

An equally potent agent of public health education is the public health nurse, or health visitor. I do not propose to describe her work in the promotion of infantile and maternal hygiene, in the supervision of the home-life of the tuberculous patient, in the investigation of home conditions of school-children, and of hospital patients, and in other branches of practical work in personal hygiene. These activities are well known to you. In remote country districts this work is often combined with that of actual sick nursing in acute or chronic illness; and emphasis needs to be laid on the high value of such nursing, not only because of the immediate benefit thus received, but even more because—the minds and hearts of patients and relatives being specially receptive in these circumstances—of the indirect hygienic value of such work in the future life of the family thus influenced. In this instance there can be no doubt of the value of instruction to adults. It is demonstrative, not didactic, in character; and this is the special aid from which most immediate benefit may confidently be expected.

In this connection I may mention the special clinics and hospitals organised by the American Public Health Service, which have been started in a few centres in Virginia and in other States for the treatment of trachoma. Here is a widely-spread damaging disease of the eyelids, a frequent cause of partial or complete blindness, of malnutrition, of dwarfed growth, and of industrial dependence, which can be cured under proper treatment, and which can be prevented from spreading from patients who have learnt the nature and method of control of its infection. A better illustration of the synthesis of treatment and prevention could scarcely be quoted.

The teaching of these paragraphs might be summed up in the statement that one of the best and often the best means for preventing the spread, as also the continuance of illness, is to give skilled help to the patients suffering from illness.

In what I have said I have ventured to emphasise the importance of certain guiding motives of life, if the world's problems, and our local and State, political, and sanitary problems are to be solved. These problems are immensely complex; civilisation has become an intricate entanglement of ten thousand conflicting interests. Strong individualities, personal and class interests, corruption and

**Public Health Education—(*cont.*).**

graft—the more dangerous the less direct and gross they are—and faddism of various kinds, render the steps of progress slow and halting. The correctives which will enable us to go right are loyalty and love for one another, arising out of the fulfilment of the second and great Commandment.

A university can become, and is already in some measure, the centre around which this becomes possible. The students in it are still in the cartilaginous stage of development of habits and principles, and may be expected, in large measure, to take the form of their environment. Much of the hope of the future depends on every member of the community realising the outlook of the Arab, who when asked which of his children he loved best said:—

The babe until he is grown,
The absent one until he returns,
The sick one until he is well.

No superman is needed for this socially essential work, but just the ordinary man trusting and trusted by his fellows, one who has substituted the impersonal for the personal outlook. And that after all is the essence of Christianity, to which as the ages pass we gradually approximate. On the large scale the task in the past has not been tried and failed; it has been found difficult and not tried. But we must try it; it is the only bulwark against anarchy; the only lever by which humanity can be uplifted.

---

The quarantine officer at the Port of San Francisco recently made a report of an unusual and interesting incident in connection with the fumigation of the steamship *Bali*, bound in from Java. Of the total number of approximately 100 rats destroyed as the result of fumigation 89 were destroyed by the fumigation of the four lifeboats. If the lifeboats are furnished with a tight covering they can be fumigated, and this is the practice that is often resorted to, allowing an excess percentage of gas to compensate for leakage, but where fumigation is a physical impossibility lifeboats can be flooded with water. Rats are prone to frequent lifeboats of a vessel to a greater or less degree because of a water supply that can be found there when not available in other parts of the vessel.

---

The prosperity of any municipality is directly dependent upon the health conditions which prevail there. Inefficient control of communicable disease costs the taxpayers inestimable and unnecessary sums of money in doctors' bills, nursing service, and loss of time from work, not to mention the worry in connection with sickness in a family. No expenditure of public funds gives a greater return for the money spent than that used for public health purposes.—*Report of Board of Health, Montclair, New Jersey.*

---

The Medical Research Council, in consultation with the Ministry of Health, have appointed the following Committee for the investigation of the causes of dental decay: Professor W. D. Halliburton, M.D., F.R.S. (Chairman); Norman G. Bennett, M.B.; Leonard Colebrook, M.B.; J. M. Hamill, O.B.E., M.D.; Sir Arthur Keith, M.D., F.R.S.; Mrs. Edward Mellanby; J. Howard Mummery, C.B.E., M.R.C.S.; C. J. Thomas, M.B.

### THE STRATFORD SCHEME.

In different parts of the country variations of the well-known and so-called insurance schemes associated with the Brighton and Oxford hospitals are being tried successfully. An interesting instance, which deserves to be better known, is that started in connection with the Stratford-on-Avon Hospital.

A hospital maintenance fund has been formed under the chairmanship of Mr. Trevor Matthews, and the following are the chief points found in the committee's report. In April 1920 the committee was formed to raise funds for the hospital, particularly from the wage-earners of the district. The houses of Stratford were thoroughly canvassed for regular subscriptions, "based on the payment of a halfpenny per 10*s*. of wages earned." Such a contribution entitled the donor and his dependants to "free maintenance at the hospital, and to free medical and surgical attendance, according to the hospital's rules." In July 1920 the contributions were received. It was decided that branches of the fund were to be formed in all the parishes served by the hospital, and during the past year meetings have been held in nearly forty villages, and many branches have been established. The results, the committee states, have exceeded its most sanguine expectations, and "from the first the scheme has been taken up with much enthusiasm everywhere." The hospital committee has made variations in the rules to meet the new situation, and the amended rules make the chairman of the committee of the fund an *ex-officio* governor, and allow the committee to nominate four members, out of a total of sixteen, to the board of management. Three of these four are also to serve upon the house committee of the hospital. A finance committee of six has also been formed, three of whom will be nominated by the committee of the fund. All in-patients, except regular contributors to the maintenance fund, or other approved fund, residing in the parishes where such funds exist, shall pay a minimum of 2*s*. a day towards the cost of their maintenance, and also present an in-patient ticket.

The balance sheet of the maintenance fund shows that a sum of £2,000 was paid to the hospital during the year ended June 30 last. It is interesting to add that the fund has also received handsome sums from various outside helpers, such as the Stratford Sportsmen's Fund Committee, which gave £125.

If the district of which Stratford-on-Avon is the hospital centre be considered, and compared with such different neighbourhoods as Brighton and Oxford, it is plain that the principle underlying the "Oxford" and "Sussex" schemes is of general application, though each district may vary its form to suit its local needs. The note of the report here summarised is cheerful and inspiring, and should encourage other parts of England to make a similar experiment.

